# White out hemithorax secondary to salivary gland type of lung cancer with metastasis in liver and bone: a case report

**DOI:** 10.1186/s13256-024-04607-y

**Published:** 2024-06-23

**Authors:** Padma V. Badhe, M. Sivakumar, Swastika Lamture, Sanika Patil, Khushboo Tekriwal, Khalid Ahmad Mohammad

**Affiliations:** Department of Radiology, Seth GSMC and KEM Hospital, Mumbai, 400012 India

**Keywords:** Adenoid cystic carcinoma, HRCT lung, Bronchial cut-off sign, Submucosal, Cribriform, Mucoepidermoid carcinoma

## Abstract

**Background:**

Salivary gland-type lung carcinomas are uncommon neoplasms of the lung, representing less than 1% of all lung tumors. The two most common among them are adenoid cystic carcinoma and mucoepidermoid carcinoma. Although they usually have an indolent behavior, adenoid cystic carcinomas can be more aggressive, with 5-year survival as low as 55%. Very few cases are reported in literature. We report a similar rare case of salivary gland type lung carcinoma that presented for the first time with unilateral opacification of left hemithorax.

**Case presentation:**

A 38-year-old man of North Indian origin, who was a a nonsmoker, presented with complaints of shortness of breath and cough for 1 year, which has increased in the last 2 months and was associated with significant weight loss. A frontal radiograph of the chest and computed tomography of the chest were performed, which showed a mass in the left upper lobe of the lung with its epicenter in the left main bronchus. A bronchoscopic guided biopsy was performed, and histopathology confirmed the diagnosis of lung carcinoma of salivary gland type (adenoid cystic carcinoma). There was invasion of major vessels, hence the patient was offered and started on palliative management instead of surgical treatment.

In spite of palliative management of two cycles of chemotherapy and radiotherapy, the patient succumbed to the disease within 2 months from the time of diagnosis.

**Conclusion:**

Lung carcinoma of the salivary gland type (especially adenoid cystic carcinoma) usually presents at a later stage. The resectability of the tumor depends on the involvement of the surrounding major vessels. Interestingly, these cancers have no association with smoking. The prognosis depends on the extent of the disease at the time of diagnosis. Hence, imaging plays a major role in deciding the further plan of management.

## Introduction

Primary salivary gland-type tumors of the lung occur primarily in the central airways and originate from the submucosal glands. These include adenoid cystic carcinomas (ACCs), mucoepidermoid carcinomas (MECs), and epithelial−myoepithelial carcinomas (EMECs) [1]. Most ACCs in the lung occur in the distal trachea or mainstem bronchi, a peripheral or segmental location are uncommon (10% of cases). Lung ACCs tend to have submucosal extension within the airway [2, 3]. MECs most commonly occur in segmental bronchi compared with the trachea or main bronchi, and appear as sharply marginated, either ovoid or lobulated, intraluminal nodules that adapt to the branching features of the airways [4]. A much less common entity is primary pulmonary EMEC, which appears to be a low-grade malignant neoplasm with a low risk of recurrence after surgical excision [5, 6].

Primary salivary gland-type tumors of the lung differ from the more common types of lung cancer (adenocarcinoma and squamous cell carcinoma) in that the former tend to occur in younger patients [7–9] to affect the central airways and to have a more indolent nature [10, 11]; however, staging and treatment of primary salivary gland-type tumors does not differ from the staging and treatment of adenocarcinoma or squamous cell carcinoma. Unlike staging of adenocarcinoma and squamous cell lung cancers, which are predominantly affected by positive nodal and metastatic disease, staging of the primary salivary gland-type tumors of the lung are usually affected most by primary local tumor invasion. Failure to identify tumor growth along the airways or in the submucosa leads to incorrect delineation and staging of the primary salivary gland-type tumors of the lung, which impacts surgical and radiation planning. Inadequate planning can affect patients negatively because local recurrence is likely if complete surgical resection is not achieved [11].

### Case presentation

A 38-year-old man of North Indian origin, who was a nonsmoker, presented with complaints of shortness of breath grade I (NYHA, New York Heart Association) and cough for 1 year, which was initially dry and was later associated with mucoid expectoration for the last month. The patient had a history of 5–6 episodes of hemoptysis per day with 5–10 cc/day in the last month. The patient had a history of approximately 8 kg of weight loss in the last 2 months associated with loss of appetite. The patient also complained of lower back pain in the last 2 months. He had no history of tuberculous contact or tuberculosis.

**Fig. 1:**
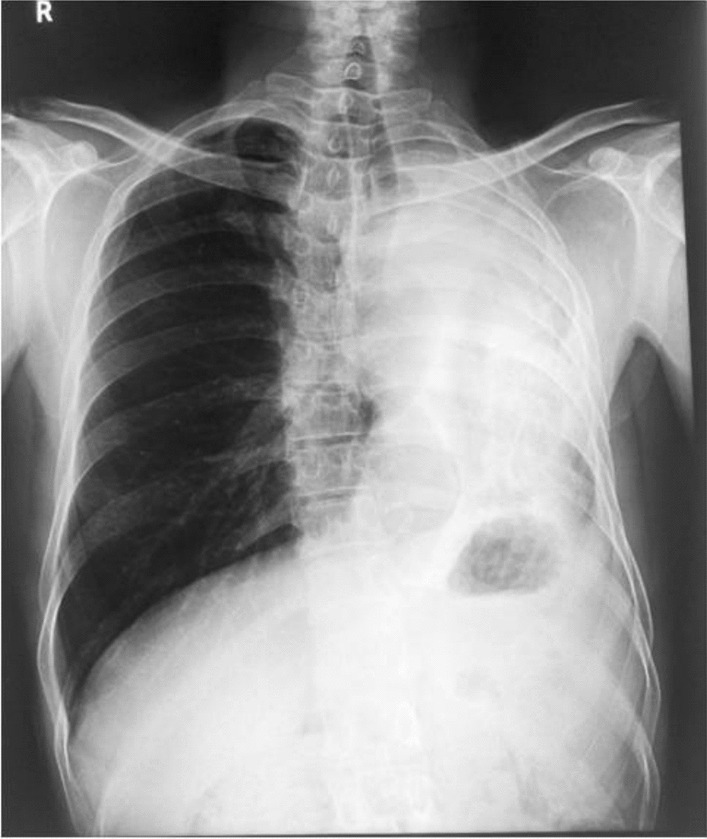
Chest radiograph shows heterogeneous opacification of the left hemithorax with an ipsilateral shift of mediastinum and loss of left lung volume. There is an elevation of the left hemidiaphragm with crowding of ribs and hyperinflation of the right lung

On physical examination, he had normal vital signs and was cachectic. On respiratory examination, he had decreased air entry on the left side. A chest radiograph revealed a heterogeneous opacification of left hemithorax, ipsilateral shift of mediastinum, elevation of left hemidiaphragm, crowding of ribs on left, and hyperinflation of the right lung (Fig. 1). These features were suggestive of volume loss of the left lung. There was a bronchial cut-off sign on the left. There was minimal blunting of the left costophrenic angle. The right costophrenic angle was normal.

A differential diagnosis of bronchogenic carcinoma and tuberculosis was considered.

**Fig. 2:**
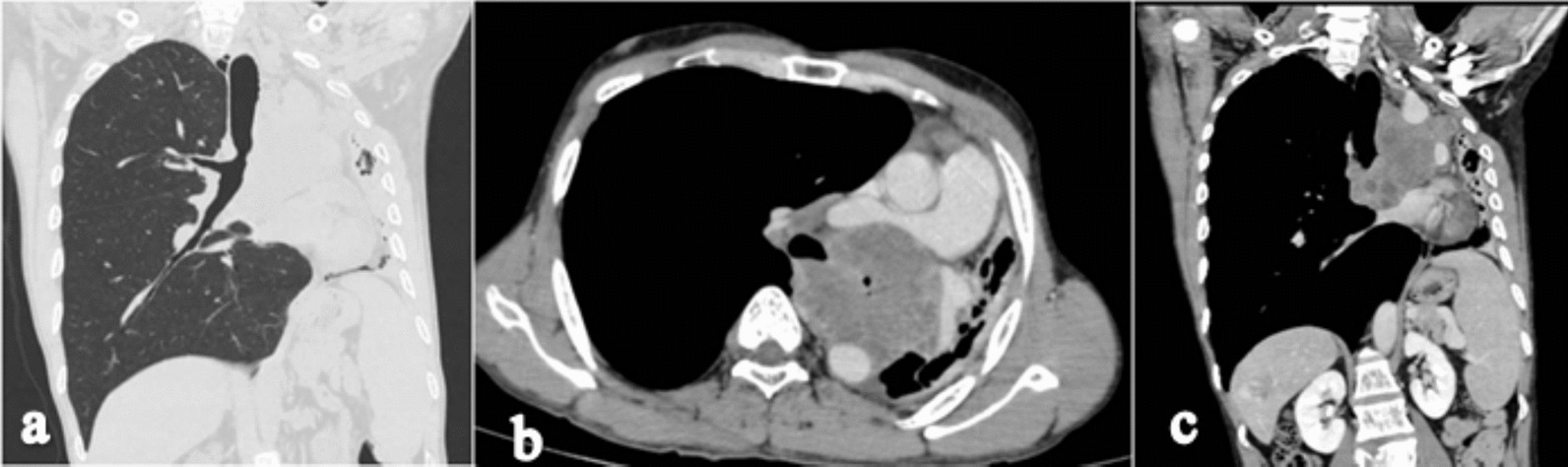
High-resolution computed tomography chest coronal section (**a**) shows cut off of the left main bronchus, loss of left lung volume with hyperinflation of the right lung, and herniation across the midline. Contrast enhanced computed tomography chest axial section (**b**) shows a well-defined lobulated heterogeneously enhancing mass in the left upper lobe with its epicenter in the left main bronchus with complete occlusion of the left main bronchus. Contrast enhanced computed tomography chest coronal section (**c**) shows a complete collapse of the left upper lobe with a shift of mediastinum and compensatory hyperinflation of the right lung with minimal left pleural effusion

**Fig. 3:**
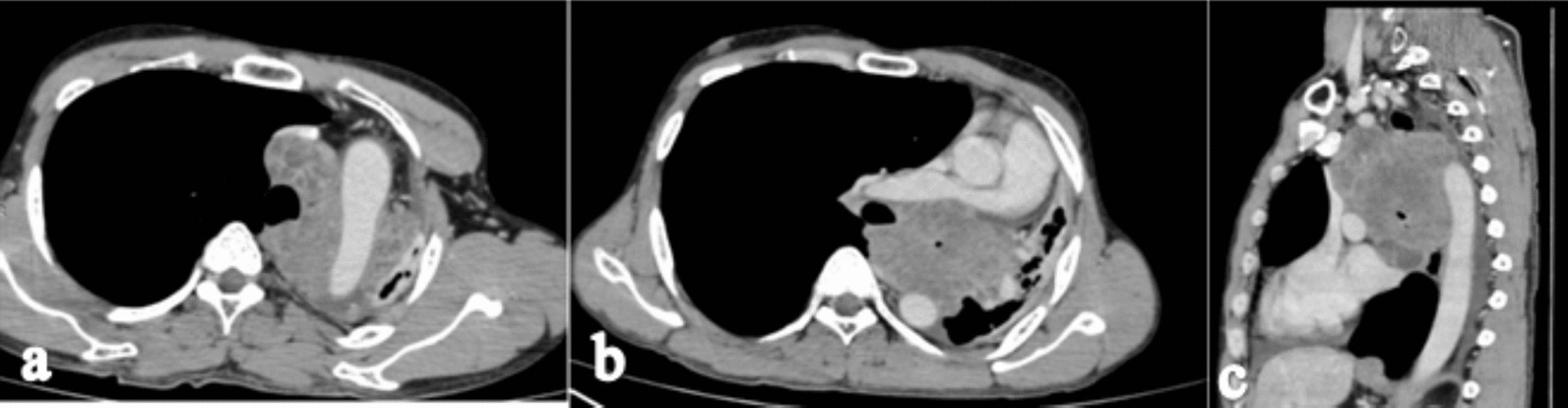
Contrast-enhanced computed tomography axial section shows complete encasement of the arch of aorta (**a**), 150° encasement of the right pulmonary artery with narrowing (**b**), and sagittal section shows > 180° encasement of descending aorta (**c**)

High-resolution computed tomography (HRCT) of the chest in the lung window shows cut off of the left main bronchus with a soft tissue lesion in the upper lobe of the left hemithorax (Fig. 2a). Contrast enhanced CT (CECT) of the chest axial section shows a well-defined lobulated heterogeneously enhancing mass of size 9 × 8 × 7 cm in the left upper lobe with its epicenter in the left main bronchus with complete occlusion of the left main bronchus (Fig. 2b). CECT chest coronal section shows a complete collapse of the left upper lobe with an ipsilateral shift of mediastinum and compensatory hyperinflation of the right lung with minimal left-sided pleural effusion (Fig. 2c). There was no mediastinal lymphadenopathy. CECT axial section shows complete encasement of the aortic arch (Fig. 3a), 150° encasement of the right pulmonary artery with its narrowing (Fig. 3b), and sagittal section shows > 180° encasement of the descending aorta (Fig. 3c).

**Fig. 4:**
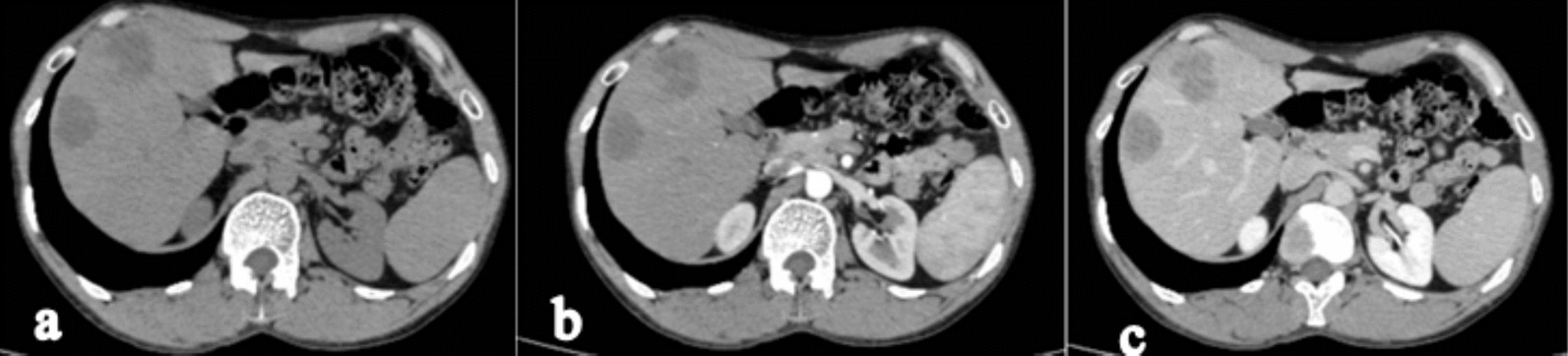
The contrast enhanced computed tomography axial section of the abdomen (**a** Plain, **b** arterial phase **c** venous phase) shows two hypoenhancing lesions in segment VI and VIII of the liver, the largest of which is 3.8 × 4 cm in size in segment VIII, suggestive of metastasis in the liver

The CECT axial section of the abdomen shows two hypoenhancing lesions in segments VI and VIII of the liver, the largest of which is 3.8 × 4 cm in size in segment VIII, suggestive of metastasis in the liver (Fig. 4a–c). However, a positron emission tomography scan was not done.

**Fig. 5:**
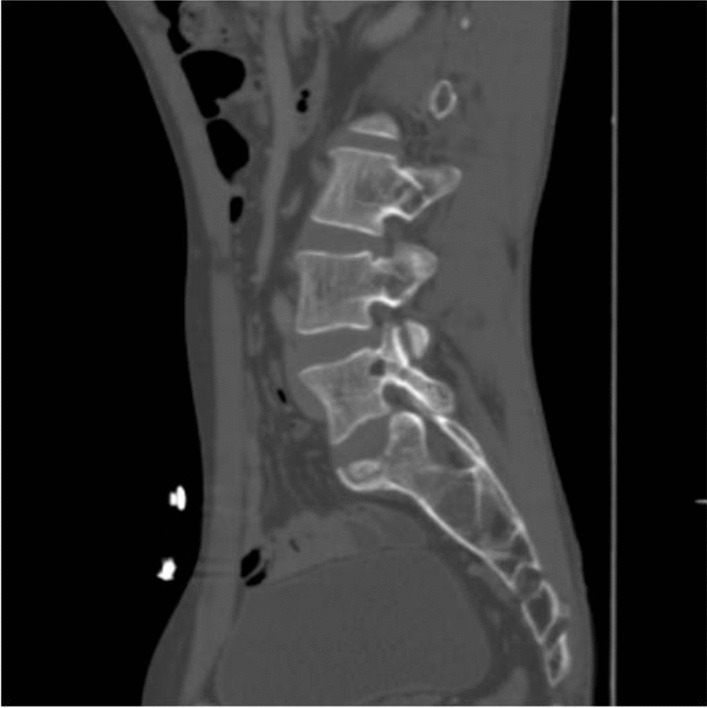
The bone algorithm of the lumbar spine in the sagittal section shows a focal lytic lesion in the left pedicle of the L5 vertebra suggestive of bony metastasis

The bone algorithm of the lumbar spine in the sagittal section shows a focal lytic lesion in the left pedicle of the L5 vertebra suggestive of bony metastasis (Fig. 5).

**Fig. 6:**
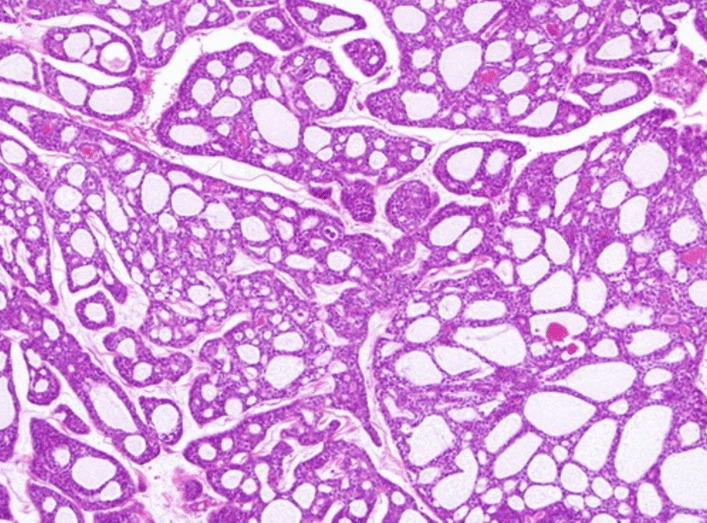
Bronchoscopic guided biopsy shows nests of cells with cylindromatous microcystic spaces suggestive of a cribriform pattern of adenoid cystic carcinoma of the salivary gland type

Bronchoscopic guided biopsy was done, which showed nests of cells with cylindromatous microcystic spaces that contained basophilic mucoid material suggestive of a cribriform pattern of high-grade adenoid cystic carcinoma (ACC) of the salivary gland type (Fig. 6). Immunohistochemistry was not done. There was involvement of major vessels, hence the patient was offered and started on palliative management of chemotherapy and radiotherapy instead of surgical treatment. After a total of two cycles of chemotherapy and two sessions of radiotherapy, the patient’s hemoglobin dropped from 11.2 gm/dl to 8.6 gm/dl. He had further loss of appetite and became more emaciated. The patient succumbed to the disease within 2 months from the time of diagnosis.

## Discussion

Adenoid cystic carcinoma (ACC) is the most common type of lung cancer of salivary gland origin in the central airway [12]. ACC commonly occurs in the fourth and fifth decades of life and has an equal sex distribution [12]. Metastases are very unusual, with recurrence more often being local [13]. ACC tends to occur in the central airways, such as the trachea, main bronchus, or lobar bronchus, a peripheral or segmental location is uncommon [12, 14, 15]. These tumors tend to have submucosal extension and manifest with circumferential and infiltrative growth [12, 14, 15]. ACC tends to have an intact epithelium and a smooth contour, because of its submucosal origin [12, 14]. The cephalocaudal extent of the tumor requires evaluation with three-dimensional (3D) or multiplanar reconstruction because it is underestimated with CT [16]. The optimal management of ACC of the lung is surgical resection, which has favorable outcomes. To control residual disease and recurrences, radiotherapy is indicated for unresectable tumors and incomplete resection. So, imaging plays a major role in evaluating the extent of the tumor, major vessel invasion, and deciding the line of management. Most ACCs should be distinguished from bronchogenic carcinoma [for example, squamous cell carcinoma (SCC)], carcinoid tumors, and benign airway tumors because they manifest as central airway tumors. ACCs that occur in the central airways and show avid FDG uptake may be difficult to differentiate from bronchogenic carcinoma, particularly SCC [12] Primary pulmonary ACC can have three main architectural growth patterns, in keeping with ACC of the salivary glands. The most common pattern is the cribriform or cylindromatous pattern characterized by nests and islands of tumor cells with well-circumscribed luminal spaces containing mucinous or basement membrane-like material. The tubular pattern consists of cells forming small, scattered gland-like spaces with wide lumina lined by cuboidal cells two to three layers thick. Finally, the solid variant is composed of nests of cells with only rare intercellular spaces, the absence of cystic areas, and a limited stromal matrix. Cytologically, tumor cells consist of bland cells with round nuclei, dense chromatin, and scant eosinophilic or amphophilic cytoplasm. There is no significant pleomorphism or mitotic activity and areas of necrosis and hemorrhage are rare. The solid variant may show an increased mitotic count, and prominent perineural, bronchial mucosal, and cartilage invasion may be seen with all patterns.

Mucoepidermoid carcinoma (MEC) in the tracheobronchial tree is rare, accounting for only 0.1–0.2% of all pulmonary malignancies [17, 18]; it is thought to arise from the minor salivary glands in the tracheobronchial tree [19]. This tumor is classified as either low-grade or high-grade on the basis of histologic criteria [9]. Low-grade MEC shows minimal or no mitoses, nuclear pleomorphism, or necrosis within the tumor [18]. High-grade tumors show increased mitoses (more than four per ten high-power fields), nuclear pleomorphism, hyperchromasia, and cellular necrosis [9, 18, 20]. MEC has been reported to occur in patients from 4 to 78 years of age, nearly one-half of whom are under 30 years old [9, 21]. Most MECs occur in the lobar or segmental bronchi rather than the trachea or main bronchi and manifest at CT as an intraluminal nodule adapting to the branching features of the airways [12, 18]. When the tumor is in the segmental bronchi, obstructive pneumonia, atelectasis, and mucus plugging are frequently associated findings. MECs have been reported to show variable amounts and patterns of FDG uptake [12]. Mild FDG uptake was found in low-grade MECs, while high and homogeneous FDG uptake has been reported in high-grade MECs [9]. As with ACCs, MECs in the central airways with high FDG uptake may be difficult to differentiate from bronchogenic carcinoma, especially SCC [12].

Primary pulmonary epithelial–myoepithelial carcinoma (EMEC) is the least common type of lung cancer of salivary gland origin. They are well-circumscribed but nonencapsulated polypoid endobronchial lesions with an average size of 3.2 cm. The lesions are often covered by intact bronchial epithelium and on the cut surface have a tan or white color. On low-power microscopic examination, the tumors have a striking biphasic appearance with duct-like structures that are formed by an inner layer of epithelial cells and a surrounding outer layer of myoepithelial cells. High-power examination reveals that the epithelial cells are flat, cuboidal, or columnar-shaped and contain eosinophilic cytoplasm and round to oval nuclei. Significant cytologic atypia or mitotic activity is not a common feature. The myoepithelial layer is composed of polygonal cells with eccentric nuclei, clear cytoplasm, and indistinct cell borders. Brightly eosinophilic, sometimes colloid-like deposits can fill the ductular lumina and the stroma can show fibrotic or myxoid changes. Some cases may show more solid tumor patterns or foci of squamous metaplasia.

## Conclusion

Salivary gland-type lung cancers are a group of low-aggressive entities, usually present at a later stage with a higher tendency of recurrence/metastasis. Intensive clinical, radiological, and pathological examinations are essential to estimate risk stratification and management. The prognosis depends on the extent of the disease at the time of diagnosis. Hence, imaging plays a major role in deciding the management plan.

## Data Availability

The datasets used and/or analyzed during the current study are available from the corresponding author upon reasonable request.

## Abbreviations

ACCs: Adenoid cystic carcinomas
MECs: Mucoepidermoid carcinomas
EMECs: Epithelial-myoepithelial carcinomas
SCC: Squamous cell carcinoma
CT: Computed tomography
HRCT: High resolution computed tomography
CECT: Contrast enhanced computed tomography
